# Impact of an influenza information pamphlet on vaccination uptake among Polish pupils in Edinburgh, Scotland and the role of social media in parental decision making

**DOI:** 10.1186/s12889-020-09481-z

**Published:** 2020-09-10

**Authors:** K. Bielecki, J. Craig, L. J. Willocks, K. G. Pollock, D. R. Gorman

**Affiliations:** 1 Public Health and Health Policy, NHS Lothian, 2-4 Waterloo Place, Edinburgh, EH1 3EG UK; 2 Population Health Department, NHS Health Scotland, Edinburgh, UK; 3 School of Health and Life Sciences, Glasgow Caledonian University, Glasgow, UK

**Keywords:** Influenza, Vaccine, Immunisation, Hesitancy, Polish, Migrants, Uptake

## Abstract

**Background:**

In Edinburgh, Scotland, lower influenza vaccine uptake has been observed in primary school children in the Polish community.

**Methods:**

To address this disparity, the Polish-language version of the NHS Health Scotland influenza information pamphlet was updated and distributed in 2018 to all identified Polish pupils attending three pilot schools. The impact of the revised pamphlet was evaluated by examining changes in vaccine uptake in these schools as compared to a control group of schools, and a questionnaire was issued to all Polish parents in the pilot schools to explore their opinions of the pamphlet and preferred sources of immunisation information.

**Results:**

On average uptake was 7.4% (95% CI 1.0–13.8%, *p* < 0.05) higher in the three pilot schools in which the Polish-language pamphlet was distributed (28.7%) than control schools (21.3%). The questionnaire feedback was that 37.3% of respondents felt better-informed about the influenza vaccine following the pamphlet. The respondents reported that the most important information source in deciding whether to vaccinate is previous experience. Healthcare professionals were ranked lower in importance when making a decision. Parents, who refused consent (*n* = 65) were more likely to source information from social media, friends and family, and Polish websites compared with those who consented (*n* = 45).

**Conclusions:**

These findings suggest that issuing new Polish health literature was associated with a large increase in consent form return rate and a modest increase in uptake of the influenza vaccine by Polish pupils in the pilot schools. Social media and Polish websites were found to have a greater influence over Polish parents’ decision to immunise than UK healthcare staff and health authority information. Intensive effort is required to encourage parents towards information sources where more accurate pro-vaccination messages can be promulgated by national health services and independent expert groups. The role of social media for migrant communities requires careful consideration, especially for vaccine programmes not delivered in their country of birth.

## Highlights

- Rise in nasal influenza vaccine uptake in all ethnic groups in studied cohorts
- Return of the consent forms increased considerably in Polish pupils in 2018
- Return of consent was lowest in Polish which continue to have highest refusal rate
- The Polish pamphlet did not have significant effect on consenting rate to influenza vaccination
- After pamphlet intervention, Polish parents felt overall better informed
- Written vaccine information (pamphlets) may not be as effective as online material
- Prior experiences were top factor in Polish parents’ decision to consent vaccination

## Background

Vaccine hesitancy was identified by the WHO in 2019 as one of the top 10 threads to global health [1]. Worldwide, more than 140,000 people died from the vaccination preventable disease measles, as cases surged globally, amidst devastating outbreaks in all regions [2]. The recent ‘State of Vaccine Confidence in the EU 2018’ report showed that Poland has experienced the largest decrease in vaccine confidence of all EU countries between 2015 and 2018 [3]. The issues are complex and can be partially attributed to anti-vaccine propaganda, which has had a prominent presence in the Polish media [3, 4]. In Poland, there has been a gradual increase in mandatory vaccine refusals, from 4893 in 2007, to 23,147 in 2016, and uptake of childhood immunisations is slowly declining, as illustrated by the decrease in uptake of the first dose of the mumps, measles and rubella (MMR) vaccine; 98% in 2007 to 92% in 2019 [5]. This has resulted in a steady increase in the number of measles cases in Poland, with 188 cases in April 2019, compared to 19 in May 2018 [6]. Currently, the largest group of economic migrants in Scotland is from Poland, with 87,000 Polish nationals now living in the country [7]. The immunisation calendar in Poland differs from that of the UK as it contains compulsory vaccinations, which are paid for by the state, and recommended vaccinations, such as the influenza and human papillomavirus (HPV) vaccines, which must be purchased on behalf of the patient [8]. In Scotland, all vaccinations are voluntary and costs are covered by the National Health Service (NHS), including the influenza vaccination for risk groups, given intranasally to school-aged children. In Poland, the purchased vaccine is administered either intramuscularly or intradermally, depending on the type [9].

Edinburgh and the surrounding region has a population of almost 900,000 and has experienced a downward trend in uptake within several vaccination programmes in recent years [10]. Previously, we found that nasal influenza vaccine uptake was 25.0% in Polish children, compared to 70.7% in white British and 60.9% in other identified ethnic minorities [11]. This is in line with low uptake rates recorded in Poland, where out of 308 parent respondents, only 14.3% reported vaccinating their child for influenza [12]. Uptake of the influenza vaccine in the general population in Poland is low at 3.4% [13]. These studies of reduced uptake in the Polish community, accompanied by many anecdotal reports of Polish migrants NHS healthcare staff about the UK vaccination programme, indicate uncertainty and concern among the Polish community [LW personal communication].

This was shown in our recent study that highlighted Polish parents’ concerns about influenza vaccination’s side effects, new vaccines in general, and the accuracy of professional vaccination advice and information sources [14]. Utilising information gathered from qualitative research undertaken with the Polish community in the spring of 2018 [15], the research team worked with NHS Health Scotland to amend the Polish version of their nasal influenza information pamphlet for primary school pupils for the 2018 influenza season. We hypothesized that distributing the pamphlet would increase the consent form return rate for influenza vaccine from the Polish pupils in the three pilot schools, and ultimately result in an increase in uptake.

## Methods

The study is comprised of two analytical components. The first is the assessment of impact of the Polish-language pamphlet on the uptake of nasal influenza vaccination among Polish pupils in the pilot cohort compared to the previous year, as well as a comparison of the pilot with a control group. The second is the assessment of how much better parents of Polish pupils feel informed with respect to the influenza vaccination programme.

In Scotland, parents of primary school children are given information and consent forms for the annual influenza vaccination programme at the start of the school term in August, and the nasal influenza vaccines are administered at schools during the school day between September and December.

Previous versions of the Polish translation of NHS Health Scotland Influenza pamphlet were a direct translation from English to Polish. The bilingual researcher, who facilitated the focus groups with Polish mothers about their perceptions of the vaccination programme in the UK in the spring of 2018 [15], worked with the Polish NHS translators with NHS Health Scotland, the publisher of the pamphlet, to revise the Polish-language pamphlet to include various elements raised in the focus group, that a new Polish migrant might question, such as: what is the influenza programme, what are the differences between the UK and Polish vaccination schedules, more information about adverse side effects, and recipients of the vaccine. The summarised table of contents of the pamphlet can be found in the supplementary files. The updated Polish influenza pamphlet was included alongside the general influenza information package (which contains a letter, information pamphlet and consent form, all in English) for all identified Polish pupils attending the three Edinburgh schools. These were the same three Edinburgh schools that were identified in previous influenza uptake research in 2017 [11] as the three schools with the largest Polish pupil population. In total, 855 pupils attended the three pilot schools, with 387 pupils (45.3%) identified as Polish. Pupils are expected to deliver the influenza vaccine information and consent forms to their parents via their schoolbags and return the signed forms to school. Each school in Edinburgh also received a media package to help promote the campaign (e.g. email and text message reminder templates and schedules, promotional posters, etc.) and were encouraged to signpost parents to the NHS Inform website for further information [16].

To evaluate the new Polish-language impact on influenza vaccination uptake, the uptake in 2018 was compared to the two previous years, 2016 and 2017. Six control schools, with similar numbers of Polish pupils (*n* = 404) did not receive the updated Polish-language pamphlet in the school pack sent home with the consent form, however, the Polish-language pamphlet was available online for parents to potentially access. Influenza vaccination uptake statuses in 2016 to 2018 of the pupils from these 9 schools were acquired from the Child Health Surveillance Programme (CHSP) for pupils attending the schools in 2016, 2017 and 2018. All children in these primary schools (years 1–7), who were eligible for influenza vaccination, form the denominator for the study.

CHSP identified the children who had been vaccinated, and the stored consent forms were reviewed to determine who had refused. Cross-referencing this data with the school’s enrolment index allowed identification of pupils who had not returned a consent form.

Ethnicity and gender were determined for each child in three stages. First, the CHSP database and TRAKcare [17], the local NHS electronic health system which holds ethnicity data recorded according to census classification, were matched using each child’s Community Health Index (CHI) number (the unique personal patient identifier used by NHS Scotland). Second, extracts from the School Education Electronic Management System (SEEMIS) [18] for the nine schools were matched to each pupil to augment ethnic and gender information for those children where this was not found in CHSP or TRAKcare. Finally, consent forms were hand-searched by one Polish-speaking study author (KB) to identify any other children as being of ‘White Polish’ ethnicity through their traditional Polish names and surnames on the CHSP database, or through parental/maiden names recorded on consent forms. The pupils were clustered in three ethnic groups for analysis: UK, Polish, and Other Identified Ethnic Minorities (OIEM), as well as the Total, which included the pupils who had unknown or unrecorded ethnicities.

### Polish NHS influenza pamphlet feedback questionnaire

One week following each of the pilot schools’ vaccination sessions in the autumn of 2018, a questionnaire (supplementary file) with a covering letter in Polish was sent to parents and/or guardians of all the identified Polish pupils who had received the updated Polish influenza pamphlet. This questionnaire used in this study was developed for this study. All of the questions were the same, but the front page of the questionnaire was marked for the researchers to be able to identify whether that pupil’s parent had agreed to immunisation, had refused or had not returned their consent form.

This questionnaire was created in English and reviewed by the research team, and then translated into Polish by the Polish-speaking first author (KB). It sought to evaluate the utility of the pamphlet and explore other influences on vaccination decision-making.

Schools were encouraged to remind parents to return the questionnaires, and after 2 weeks the returned questionnaires were collected from the schools’ offices. The questionnaire responses gave qualitative information about the impact of the Polish pamphlet and the range of information sources and influences on parents’ decision making about influenza vaccination.

### Data analysis

In both the pilot and control groups, influenza vaccination uptake, refusal, and non-return rates of consent forms were analysed by ethnicity between 2017 and 2018. Percentage uptake and paired t-test was used to analyse the statistical significance. In the control group, the paired t-test was used to analyse any significant percentage change.

From the collected evaluation questionnaires, comments were translated from Polish to English by a Polish researcher (KB). All data analyses were completed on SPSS, version 25.0 (IBM, USA), and graphical representations were created using Tableau software (version 10.3, USA).

## Results

### Pilot schools cohort

**Fig. 1:**
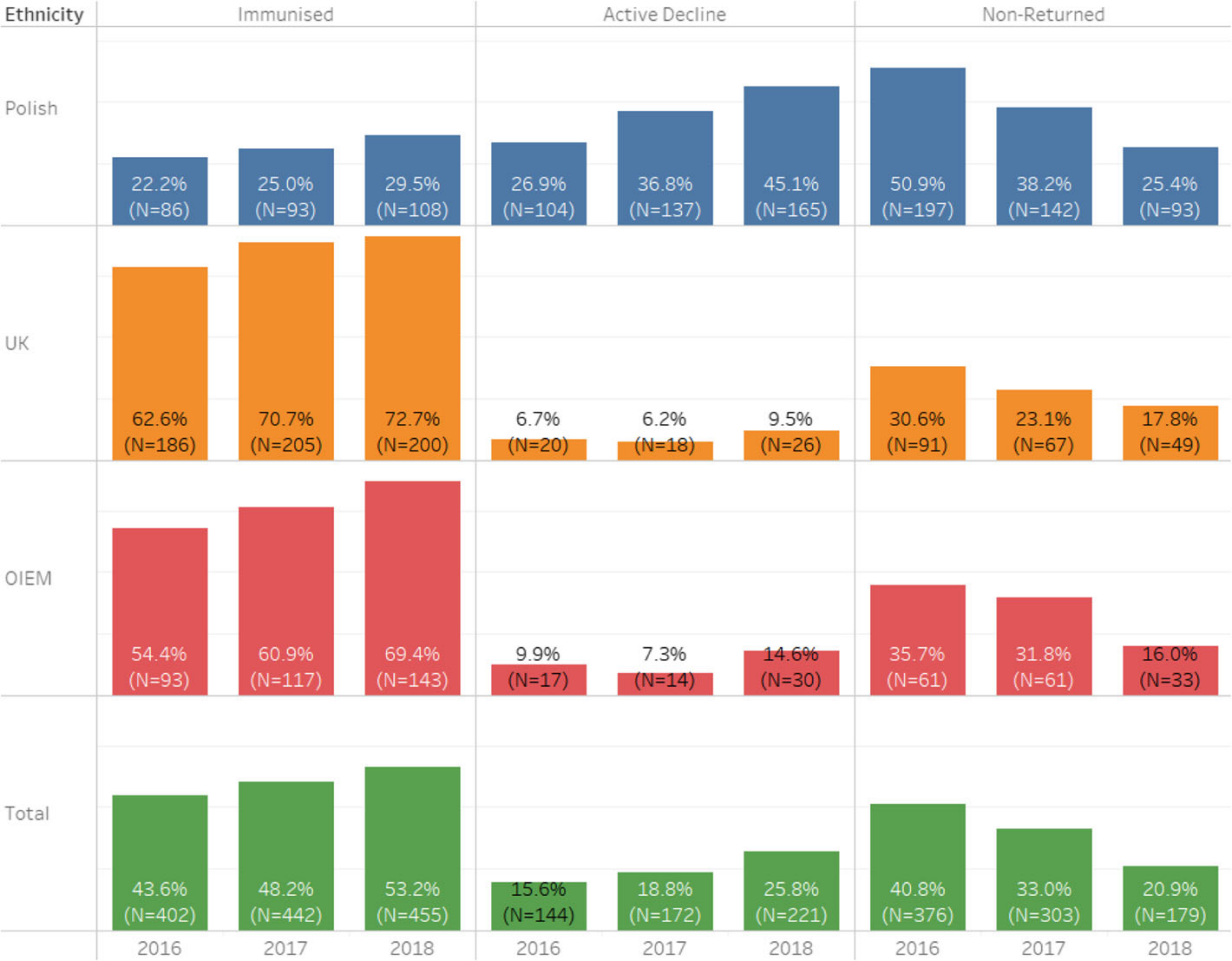
Comparing Consent Form Returns of Ethnic Groups between 2016, 2017, and 2018 in the 3 Pilot Edinburgh Schools

For all pupils in the pilot schools’ cohort, the uptake of influenza vaccine in 2018 increased by 5.0% (95% CI 3.4–9.7%, *p* < 0.05) as presented in Table 1 and illustrated in Fig. 1. The refusal rate increased overall in the schools by 7.3% (95% CI 3.1–10.9%, *p* < 0.001); Polish pupils’ refusal rate increased by 8.3% (95% CI 1.2–15.4%, *p* < 0.05), and OIEM pupils’ refusal rate increased by 7.3% (95% CI 1.1–13.5%, p < 0.05). The vaccination consent at the three pilot schools ranged 47.8 to 61.3%.

**Table 1:**
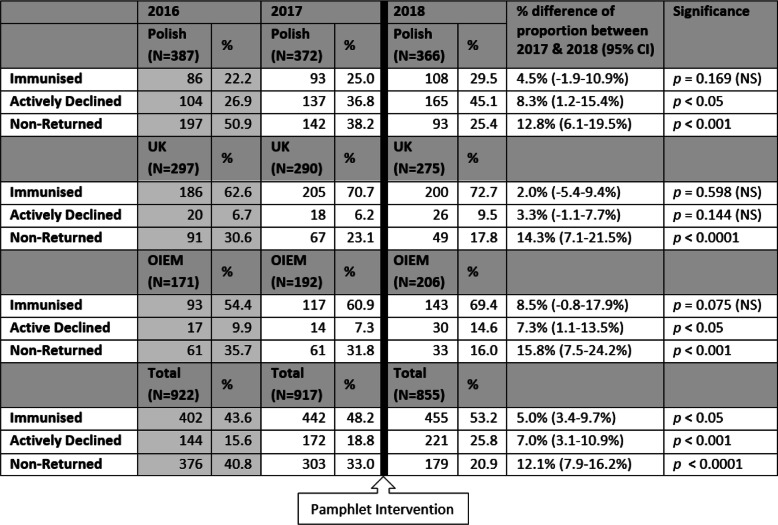
Comparing consent form returns of ethnic groups between 2016, 2017, and 2018 in three pilot Edinburgh schools

### Control schools cohort

**Fig. 2:**
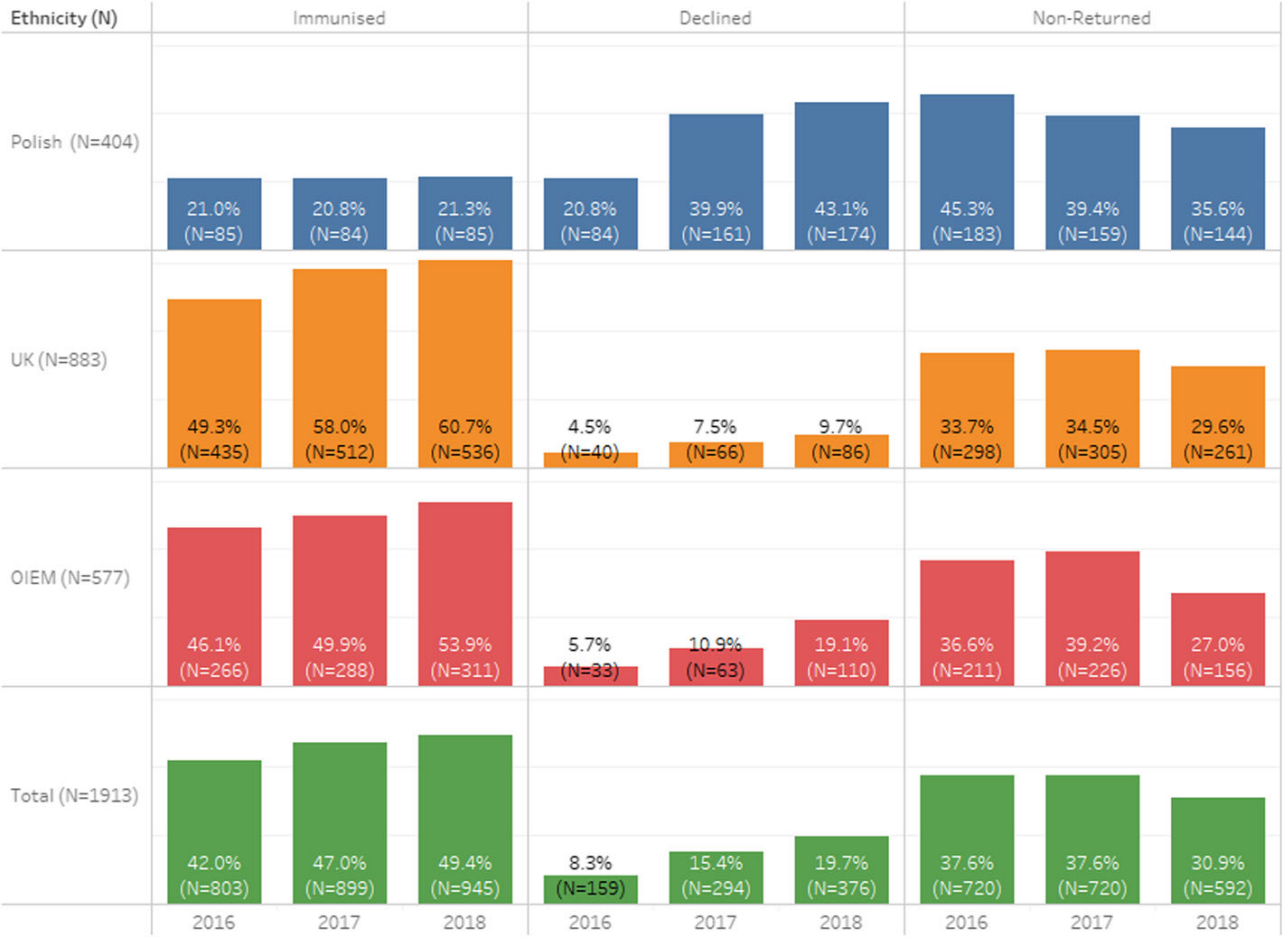
3-year trend of Influenza Vaccine Uptake Rates of Ethnic Groups of pupil cohort at six control Edinburgh schools

The pupil cohort attending the six control schools in 2018 was examined (*N* = 1913), by uptake and consent form return rates in 2016, 2017 and 2018 in Table 2 and illustrated in Fig. 2. Overall, in 2018, the vaccination uptake rates at these six schools improved by 0.5–4.0% across all ethnic groups, although none were statistically significant.

**Table 2:** 3-year trend of Influenza Vaccine Uptake Rates of Ethnic Groups of pupil cohort at six control Edinburgh schools

Ethnicity (N)	Uptake	2016		2017		2018		% Difference between 2017 & 2018 (95% CI)	Significance
**Polish (*N*** **= 404)**	**Immunised**	85	21.0%	84	20.8%	85	21.3%	0.5% (−5.1–6.1%)	*p* = 0.816 (NS)
	**Refused**	84	20.8%	161	39.9%	174	43.1%	3.2% (−3.6–9.9%)	*p* = 0.356 (NS)
	**Non-Returned**	183	45.3%	159	39.4%	144	35.6%	3.8% (−2.8–10.5%)	*p* = 0.265 (NS)
**UK (*N*** **= 883)**	**Immunised**	435	49.3%	512	58.0%	536	60.7%	2.7% (−1.9–7.3%)	*p* = 0.248 (NS)
	**Refused**	40	4.5%	66	7.5%	86	9.7%	2.2% (−0.4–4.8%)	*p* = 0.099 (NS)
	**Non-Returned**	298	33.7%	305	34.5%	261	29.6%	−4.9% (−0.5–9.2%)	*p* < 0.05
**OIEM (*N*** **= 577)**	**Immunised**	266	46.1%	288	49.9%	311	53.9%	4.0% (−1.7–9.7%)	*p* = 0.174 (NS)
	**Refused**	33	5.7%	63	10.9%	110	19.1%	8.2% (4.1–12.3%)	*p* < 0.0001
	**Non-Returned**	211	36.6%	226	39.2%	156	27.0%	−12.2% (−6.7–17.6%)	*p* < 0.0001
**Total (*N*** **= 1913)**	**Immunised**	803	42.0%	899	47.0%	945	49.4%	2.4% (−0.7–5.6%)	*p* = 0.137 (NS)
	**Refused**	159	8.3%	294	15.4%	376	19.7%	4.3% (1.9–6.7%)	*p* = 0.0005
	**Non-Returned**	720	37.6%	720	37.6%	592	30.9%	6.7% (3.6–9.7%)	*p* < 0.0001

In the control schools’ cohort, the number of parents refusing the influenza vaccination increased by 27.9%, while the number of parents consenting rose by only 5.1%. Among the Polish pupils in the control group, there was a 1.2% increase in the number of vaccinated pupils (one more pupil than the previous year) and an 8.1% increase in the number of refused forms.

### Comparing pilot and control schools cohorts

Table 3 compares the influenza vaccination consent form return history of the Polish pupils in the pilot and the control schools, who attended in 2018 to their previous status in 2017. The only significant finding was the 7.4% (95% CI 1.0–13.8%, *p* < 0.05) difference in proportion between the pilot and control groups’ 2018 immunised rate, the year after the pilot Polish pupils received the revised Polish-language pamphlet.

**Table 3:** Comparing polish pupil consent form return status between pilot and control school groups

	Consent Form Status	Polish Pupils in Pilot Group (***N*** = 303)		Polish Pupils in Control Group (***N*** = 404)		Difference (%) in proportion between Groups (95% CI)	Significance
**2017 (No pamphlet)**	Immunised	76	25.1%	84	20.8%	4.3% (−19.3–10.5%)	*p* = 0.177 (NS)
	Refused	110	36.3%	161	39.9%	3.6% (−3.6–10.8%)	*p* = 0.333 (NS)
	Non-Returned	117	38.6%	159	39.4%	0.8% (−6.5–8.1%)	*p* = 0.829 (NS)
**2018 (Pamphlet distributed to Pilot Group)**	Immunised	87	28.7%	85	21.3%	7.4% (1.0–13.8%)	*p* < 0.05
	Refused	129	42.6%	174	43.1%	0.5% (−6.9–7.9%)	*p* = 0.894 (NS)
	Non-Returned	87	28.7%	144	35.6%	6.9% (0.0–13.9%	*p =* 0.053(NS)

### Polish NHS influenza pamphlet feedback questionnaire results

One week following each school’s nasal influenza vaccination date, a letter and questionnaire in Polish were sent home to parents/carers of all Polish pupils, who received the updated Polish Influenza pamphlet. The questionnaire sought feedback about the pamphlet and parents’ views about vaccinations in general. After 2 weeks, the questionnaires were collected from the schools’ offices. School A returned 46 out of 106 (43.4%), School B returned 47 out of 112 (41.9%) and School C returned 35 out of 147 (23.8%). Out of the 165 parents who had refused consent for their child’s influenza vaccination in 2018, 65 respondents replied (39.4%). Out of 108 parents who had consented to influenza vaccination, 45 replied (41.7%), and out of 92 parents who had not returned a consent form, 18 replied to the questionnaire (19.6%). In total, 128 out of 365 (37.3%) questionnaires were returned.

### Respondent demographics

Of the 128 replies, the majority of respondents were female guardians/mothers (82%). Most of the Polish parents have been living in the UK for 6–15 years (89.8%). These families predominantly speak in Polish at home (71.9%). Six pupils previously received vaccinations in Poland – four of these children received all their Polish-compulsory childhood vaccinations and two had additional immunisations in Poland. Table 4 summarises the returned questionnaire respondents’ demographics.

**Table 4:** Summary of polish influenza feedback questionnaire respondent demographics

Question	Replies	Proportion (***N*** = 128)
Gender of Responding Parent/Guardian	Male	17.2% (22)
	Female	82.0% (105)
	Rather not state	0.8% (1)
Number of Years Living in UK	2–5 Years	7.8% (10)
	6–15 Years	89.8% (115)
	16+ Years	2.3% (3)
Language Spoken at Home	English	0.8% (1)
	Polish	71.9% (92)
	Both	27.3% (35)
First Year Child is Attending School in Scotland	No	85.2% (109)
	Yes	14.8% (19)
Total Number of Children in Family	1	21.1% (27)
	2	60.2% (77)
	3 or more	18.7% (24)
Number of Children that Received Immunisations in Poland	No	95.3% (122)
	Yes	4.7% (6)

### Factors in influenza vaccine consent decision making

A total of 123 respondents replied to the following questionnaire question: “When deciding whether to accept or decline your child’s influenza vaccine, which of these sources did you consult?” where they could choose multiple sources as listed in Table 5. The responses were further analysed based on whether the parent/guardian had consented, refused or had not returned the consent form for the 2018 school influenza programme vaccine. 27.3% of consenters, 14.5% of refusers and 33.3% of non-returners selected the NHS Polish Influenza Pamphlet as a source of information. Overall, all respondents found previous experience of vaccination the most important factor in their decision making. 33.9% of respondents who refused vaccination chose to source information from Polish-language websites, while 17.7% of refusers sourced it from English-language websites. No Polish-language website was specified in the questionnaire responses.

**Table 5:** Consulted sources of information by respondents to decide whether to immunise child in 2018 for influenza, breakdown provided by consent form status

Factor	Consented (%) ***n*** = 45		Refused (%) ***n*** = 65		Non-Returned (%) ***n*** = 18		Total (%) ***n*** = 128	
Previous Experience	24	53.3%	29	44.6%	5	27.8%	57	44.5%
Family/Friends in Poland	12	26.7%	23	35.9%	5	27.8%	40	31.3%
Family/Friends in Scotland	8	17.8%	24	36.9%	7	38.9%	38	29.7%
Social Media e.g. Facebook, Twitter*	6	13.3%	28	43.1%	4	22. 2%	37	28.9%
Polish Language Websites	7	15.6%	22	33.9%	3	16.7%	31	24.2%
NHS Polish Language Flu Pamphlet	12	26.7%	9	13.9%	2	11.1%	23	17.9%
English language Websites	8	17.8%	11	16.9%	3	16.7%	22	17.2%
UK Health Visitor/GP	7	15.6%	8	12.3%	3	16.7%	18	14.1%
Polish Language TV or Radio	7	15.6%	7	10.%	3	16.7%	17	13.0%
Polish Healthcare Staff*	11	24.4%	4	6.2%	1	5.6%	16	12.5%
Other Source	3	6.7%	11	16.9%	1	5.6%	14	10.9%
English Language TV or Radio	7	15.6%	4	6.2%	1	5.6%	12	9.4%

* Significant (*p* > 0.05)

Parents who refused vaccination were significantly more likely to choose ‘social media’ such as Facebook or Twitter as a main source of information for vaccine decision-making than consenting parents, 43.1% compared to 13.3% respectively (*x*^2^ = 11.32, *p* = 0.001) Also, 24.4% of consenting parents referred to Polish healthcare staff, which was significantly higher than the 6.2% of parents who refused (*x*^2^ = 7.48, *p* = 0.006).

### Opinions of the updated NHS health Scotland polish influenza pamphlet

In total, 75 of the 128 respondents (58.6%) remembered reading the new NHS Health Scotland Polish influenza pamphlet. From those who recalled the pamphlet, 62 (82.7%) stated they read all of it, while 9 (12.0%) read some of it. When asked which parts of the pamphlet they found most useful, 22 found “All/Everything being useful and important” and three reported finding the information on side effects and risks useful in particular.

When asked if respondents felt that any information was missing, 97% believed nothing was missing or unclear. Two respondents (3%) believed more information about adverse side effects was needed. From the respondents who read the pamphlet, 40 respondents (53.3%) stated no change in how well-informed they felt about the influenza programme this year compared to other years; however, 28 respondents (37.8%) felt better informed.

## Discussion

The main feature of the Polish parents in the pilot cohort was an increase in their return rate of the consent forms by 20.7%. This was formed of 20 pupils’ parents who chose to consent this year, while over twice as many (41) chose to refuse the vaccination. This suggests that nearly two-thirds of pupils’ parents, who previously did not return consent forms in 2017, could be described as ‘passive refusers’. That is, they knew an unreturned form would mean that their child would not be immunised.

Evidence from the control school cohort shows there was a much smaller increase in the return rate of the consent forms, suggesting the intervention had some effect. Polish parents refusing the influenza vaccination in the control school cohort increased by 3.2%, while the number of parents consenting increased by 0.5% (only one pupil). This, again, may be due to a significant increase in the number of returned consent forms, suggesting that many of the non-returning parents in previous years may have also been ‘passive refusers’.

This pamphlet study is the first intervention used to increase the uptake of the influenza vaccination in the Polish community nationally in Scotland. Brief written education interventions, such as pamphlets, are the most tested interventions in literature reviews, but were previously found to have little or no impact on vaccine hesitancy [19, 20]. Previous studies on influenza pamphlets interventions found providing education intervention in the waiting room before a paediatric provider visit may help increase child influenza vaccine receipt [21] as well as higher uptake in maternal influenza vaccination uptake in pregnancy [22] however pamphlet intervention was found ineffective in increase uptake a minority group, due to lack of personalisation and authority association in a 2018 study with Aboriginal children [23]. The decision-making process of consenting to vaccination is complex, as evidenced by the feedback from the questionnaire.

The majority of respondents (58.6%) remembered reading the new NHS Health Scotland Polish influenza pamphlet, and of those who had read the pamphlet, 82.7% stated they had read all of it and a majority of those found “all” or “everything” in the pamphlet useful. A few parents left comments expressing the desire for more information about ingredients found in the influenza vaccine. This concern was also shared with the researcher in a previous qualitative study with Polish mothers [15]. The updated pamphlet provided a direct URL link to the patient information leaflet, which lists all the ingredients of the intranasal vaccination. This suggests that the respondents had not read the pamphlet carefully or did not follow up online to check. In the next version of the pamphlet, this can be improved and made easier to find.

From the questionnaire, it was revealed that parents selected “previous experience” of the vaccination 53% of the time as the most important factor in their decision-making process when deciding whether to give consent for the influenza vaccination, and this did not differ significantly by consent form status. Polish migrants if they have arrived recently in Scotland will be unfamiliar with a school-led influenza programme, as one does not exist in Poland. Influenza vaccination is not a mandatory vaccination in Poland, and costs fall on the patient. Overall, it is not a popular vaccine and uptake is very low with the influenza vaccination uptake rate remaining at a 3% threshold nationally in the past 10 years [24]. The influenza vaccination uptake rate differs widely in Poland and depends much on the age and risk group. In patients with chronic diseases, as well as the elderly, immunization coverage is higher than in the general population; however, this still remains well below the recommended level, which is the 75% uptake in key risk groups [13, 25, 26]. The attitude toward influenza infection and vaccination is different in Poland; it is not regarded as an important vaccination and there is a lack of awareness of its need - in a national survey in Poland [24, 25] important gaps in the knowledge on influenza vaccination were found in the general Polish population.

Although healthcare staff, doctors in particular, are traditionally seen as being important in shaping health behaviour, they ranked low in this sample, especially among the refusers - only 6.2% of refusing parents chose medical staff as a key source of information about vaccines. The healthcare staff role must be enhanced to increase the influenza vaccination coverage among Polish children in Scotland, and according to the results, respondents provide a potential answer, as their top source of information about vaccinations was related to social media.

Considering that the majority of respondents have lived in Scotland for several years, the fact that they consult practitioners in Poland as much as in the UK is an indication that they continue to live in a community heavily influenced by Polish norms and values. In a 2016 cross-sectional survey of parents in Poland, it was found that medical doctors often provide the basic source of information about vaccination to parents, however, 16.9% of respondents declared that information received from physicians regarding vaccinations was either incomplete or unconvincing [27]. This Polish literature confirmed that participants in Poland were less likely to seek information about vaccinations from medical professionals, and participants who used less accurate sources, were more likely to avoid vaccination.

Overall, respondents’ top six sources of information about vaccinations were related to social contact, internet-based media and previous experience. Polish language sources were more prominent than English ones. In our study, the consenting parents had the NHS Polish influenza pamphlet as the second most selected source of information, previous experience was rated highest. Social media, where users often share anti-vaccination material [28–30], was significantly more likely to be cited as an influence by refusing parents than by consenting ones.

While the internet is used by health promoting organisations for positive and informative messages, it is also a vehicle for unmoderated anti-vaccination sentiment and misinformation. The UK-focused 2019 Royal Society for Public Health report [31] found that 41% of parents surveyed had been exposed to negative messages about vaccination on social media, rising to 50% of parents of children under five. The report discusses the risk that repetition of incorrect information is often mistaken for accuracy, citing an American study [32] that revealed that even when participants were provided with prior knowledge, they could succumb to the effects of ‘illusory truth’.

Even if just a small percentage of the population is opposed to vaccinations, social media facilitates anti-vaccination connections and organisation [33] and allows “echo-chambers” which exaggerate the group to appear larger than reality. Various social media platforms provide an online space for unregulated anti-vaccination and counter-factual information, such as lengthy lectures on YouTube by Polish doctors and academics [34, 35] who are very sceptical about vaccination. As a result of the findings of this study, and recognizing the need for accurate, evidence-based information about vaccination on social media [36], NHS Health Scotland created an immunisation focused Twitter account in March 2019. Through this account, they have shared Polish-language Tweets and infographics to promote the influenza vaccine and have engaged with the Twitter accounts of several Polish community groups in Scotland, in an attempt to further reach the Polish community with accurate information about immunisations. There is also a need to increase the presence of healthcare workers on social media [36]. Another approach to explore would be the use of talking-head videos with Polish healthcare workers in Scotland promoting the influenza vaccine, to be shared not only on NHS Twitter accounts but through the social media channels of schools and Polish community groups in Scotland.

There are efforts being made by social media platforms such as YouTube and Facebook to change their policies to reduce the amount of misinformation on their sites [37, 38]. Pinterest recently changed their search engine to only provide results from major health organisations for 200 terms related to vaccines, and bar any advertisements, recommendations and commentary on those pages [39]. Efforts need to continue to bring accurate information to the top of vaccine-related searches and remove false information that can harm people [40], but the challenges around curating content online are complex, as social media websites have the challenge of verifying the veracity of each user’s post, and restricting users’ freedom of speech.

### Limitations

Ethnicity was determined for 94% of participants in the study by using multiple methods and uptake was calculated using clinical records rather than self-reporting. The NHS data systems and recording of ethnicity data and education data are robust and we believe we have attributed ethnicity accurately. We also utilised name searching of consent forms by a Polish-native researcher (KB), which is an option not available in all locations. Polish pupils, not registered as Polish and without a Polish last name, may have been categorised in the Unknown section. A name recognition software such as Onolytics can be used as an alternative, which has been validated in several studies [41].

Study participants were limited to nine schools in one area of Scotland. While we have no reason to believe that these children and parents/carers from Edinburgh are atypical of Polish migrants in general, studies in other areas would be useful to ascertain whether our findings apply elsewhere. Larger studies would allow disaggregation of our other identified ethnicities, such as other European, Asian or minority groups, which could give useful information to vaccination programmes. Following vaccination behaviour by ethnicity over a longer period would be of value in evaluation.

In future questionnaire research, it would be interesting to pose a question about preference for mode of administration in the questionnaire, to find out if the nasal component is off-putting to Polish parents, as in Poland, the influenza vaccination is not administered nasally.

There was a 4.5% increase in the number of vaccinated children in the Polish cohort following the distribution of the tailored Polish pamphlet. While this result was not statistically significant given the sample size in the study, it suggests there is a potential for the pamphlet to increase the number of vaccinations among Polish pupils with a larger sample size.

The response rate to the questionnaire was moderate, with 128 out of 365 (37.3%) questionnaires returned. This is a limitation which might have introduced bias, however there was an even response from both the parents who consented (41.7%) and those who refused (39.4%) the nasal influenza vaccination for their child. In future research, it would be of interest to delve into respondents’ previous experiences, whether it was a previous adverse event with a vaccine or past behaviour dictating current behaviour. This would aid in preparing healthcare providers to discuss with patients about these previous experiences. Moreover, as Social Media, and both English and Polish websites were listed as sources of information, it would be of use to have respondents specify what web pages and social media outlets they turn to when making vaccination decisions.

The increase in consent form return rate was not as marked in the control schools as it was in the pilot schools - this may be due to the schools’ differing adherence to the influenza education package. The increase in consent form return rate could have been influenced by proactive narrative from a nurse in the Community Vaccination Team (CVT), who led the school influenza vaccination programme at the three pilot schools. The nurse ensured schools followed the promotional campaign and sent timely emails and text message reminders to parents to return consent forms, however we have no knowledge of the level of nurse or online promotion activity in the control schools. As such, the confounding factor of the difference in nurse enthusiasm cannot be accounted for, leading to uncertainty about the effectiveness of the pamphlet intervention. The study could be repeated with a controlled level of nurse involvement.

## Conclusion

There is a slow continued rise in nasal influenza vaccine uptake in each ethnic group. The rate of non-return of the consent forms reduced considerably in 2018, but was still highest in the Polish group, which continues to have the highest refusal rate.

Given that NHS Scotland aims to obtain informed consent from parents, having the best information available about vaccination is important, and the new pamphlet is an important step to target the low uptake migrant group with tailored information. These findings suggest that the impact of the new Polish health literature was a modest increased uptake of the influenza vaccination within Polish students in the pilot schools, however social media and Polish websites were found to have a greater impression upon Polish parents’ decision to immunise their child. More needs to be done to direct parents towards robust health websites and explore the role of social media sites, such as Facebook and YouTube, where accurate pro-vaccination messages can be promulgated by public health services.

## Supplementary information

**Additional file 1 **: **Supplementary Material 1.** English Questionnaire. Questionnaire created and used in this study

**Additional file 2 **: **Supplementary Material 2.** Pamphlet Content. Table of content of the Polish School Programme Influenza Vaccination Information pamphlet created and studied in this study.

## Data Availability

The datasets used and/or analysed during the current study available from the corresponding author on reasonable request.

## Abbreviations

CHSP: Child Health Surveillance Programme
CHI: Community Health Index
CVT: Community Vaccination Team
HPV: Human papillomavirus
MMR: Mumps, measles and rubella
NHS: National Health Service
OIEM: Other Identified Ethnic Minorities
SEEMIS: School Education Electronic Management System
