# Supplementary material for: Impact of an influenza information pamphlet on vaccination uptake among Polish pupils in Edinburgh, Scotland and the role of social media in parental decision making
Source: BMC Public Health. 2020 Sep 10;20:1381. doi: 10.1186/s12889-020-09481-z (PMC7488143; doi:10.1186/s12889-020-09481-z)
Supplement: Supplementary file 1 — Additional file 1 : Supplementary Material 1. English Questionnaire. Questionnaire created and used in this study [file 12889_2020_9481_MOESM1_ESM.docx]

# Influenza Booklet Evaluation Questionnaire – English Version

*Please complete the following questionnaire and enclose in envelope provided*

**1. Are you? Please put an X in the relevant box.**

¨ Male ¨ Female ¨ Prefer not to say

**2. How long have you lived in the UK?**

¨ Less than 1 year ¨ 2-5 years ¨ 6-15 years ¨ 16+ years

**3. What language do you speak at home?**

¨ English ¨ Polish ¨Both ¨ Other

1. **How many children do you have?** ……….
2. **How many of your children were born in the UK?** ………
3. **Have any of your children had any immunisations in Poland while you were living in the UK? Please put an X in the relevant box.**¨ **Yes** ¨ **No**
4. **If yes, please give brief details:** ...............................................................................................................................................
5. **When deciding whether to accept or decline your child’s influenza vaccine, which of these sources did you consult? Please X the following choices:**

| Family/Friends in Scotland |  | The NHS Polish language influenza booklet |  |
| --- | --- | --- | --- |
| Family/Friends in Poland |  | English language websites |  |
| Social media |  | UK Health visitor / GP |  |
| Polish healthcare staff |  | Polish language websites |  |
| Polish language television or radio |  | Previous experience |  |
| English language television or radio |  | Other source |  |

**Of these, which three were the most important?**

1. ……………………………………………………….

2. ……………………………………………………….

3. ……………………………………………………….

1. **Did you read the Polish language flu booklet?**
   ¨ Y**e**s ¨ No

**If yes did you read some or all of it?**
 ¨ I read some of it ¨ I read all of it

**Which parts did you find useful?**
....................................................................................................................................................................................................................................................................................................................................

**10. Did you feel any information was missing or unclear?** ¨ **Yes** ¨ **No

 If yes, please explain what you felt was missing or unclear:**

....................................................................................................................................................................................................................................................................................................................................

**The Vaccination in Programme in General**

**11. Please put an X in the box that describes your opinion about each question (about vaccination in general):**

|  | Strongly Agree | Agree | Disagree | Strongly Disagree | Don’t Know |
| --- | --- | --- | --- | --- | --- |
| 1. Childhood vaccines are important for my child’s health |  |  |  |  |  |
| 1. Childhood vaccines are effective |  |  |  |  |  |
| 1. Having my child vaccinated is important for the health of others in my community |  |  |  |  |  |
| 1. All childhood vaccines offered by the NHS in my community are beneficial. |  |  |  |  |  |
| 1. New vaccines carry more risks than older vaccines |  |  |  |  |  |
| 1. The information I receive about vaccines from the NHS is reliable and trustworthy. |  |  |  |  |  |
| 1. Getting vaccines is a good way to protect my child/children from disease. |  |  |  |  |  |
| 1. Generally I do what my doctor or health care provider recommends about vaccines for my child/children. |  |  |  |  |  |
| 1. I am concerned about serious adverse effects of vaccines. |  |  |  |  |  |
| 1. My child/children does or do not need vaccines for diseases that are not common anymore. |  |  |  |  |  |

**12. Are there any things missing in the current NHS information about vaccination programmes?**

....................................................................................................................................................................................................................................................................................................................................

**Thank you for your time and feedback. If you have any other comments please use the space below:**

......................................................................................................................................................................................................................................................................................................................................................................................................................................................................................................
