# Supplementary material for: Impact of an influenza information pamphlet on vaccination uptake among Polish pupils in Edinburgh, Scotland and the role of social media in parental decision making
Source: BMC Public Health. 2020 Sep 10;20:1381. doi: 10.1186/s12889-020-09481-z (PMC7488143; doi:10.1186/s12889-020-09481-z)
Supplement: Supplementary file 2 — Additional file 2 : Supplementary Material 2. Pamphlet Content. Table of content of the Polish School Programme Influenza Vaccination Information pamphlet created and studied in this study. [file 12889_2020_9481_MOESM2_ESM.docx]

**Contents of Updated Polish Pamphlet**

1. Front Covering Page - Child flu vaccine: What you need to know
2. How does the Immunisation Programme work in the UK
3. Facts about the Flu
4. What is the flu?
   - Symptoms of the flu
   - The flu can be a serious illness
   - How does the flu virus spread?
5. Flu Vaccination
   - Is the vaccine safe?
   - Does the vaccine have side effects?
   - Where and when will my child get the vaccine?
   - How is the vaccine administered?
   - Are there any reasons my child should not receive the intranasal vaccination?
   - Can my child get the flu from the vaccine?
   - What if my child is sick on the day of vaccination?
   - What will happen if my child is not vaccinated?
   - Should my child have a second dose?
   - Will be child be immunised from the flu after the vaccination?
   - How long does the vaccine immunise my child?
   - What do I do if I change my mind?
   - Where can I get more information
6. Back Covering Page: Complete UK immunisation schedule

The Polish pamphlet and 28 other translated languages can be found online here: <http://www.healthscotland.com/documents/23735.aspx>
